# Adult Atlantic salmon have a new freshwater predator

**DOI:** 10.1371/journal.pone.0196046

**Published:** 2018-04-19

**Authors:** Stéphanie Boulêtreau, Adeline Gaillagot, Laurent Carry, Stéphane Tétard, Eric De Oliveira, Frédéric Santoul

**Affiliations:** 1 EcoLab, Université de Toulouse, CNRS, Toulouse, France; 2 MIGADO, Saint-Orens-de-Gameville, France; 3 LNHE, EDF—R&D, Chatou, France; University of Windsor, CANADA

## Abstract

The Atlantic salmon (*Salmo salar*) is one of the world’s most emblematic freshwater fish. Despite conservation and rehabilitation plans, populations of this species are dramatically declining due to human impacts such as habitat fragmentation, overfishing and water pollution. Owing to their large body size, anadromous adults were historically invulnerable to fish predation during their spawning period migration. This invulnerability has disappeared in Western Europe with the introduction of a new freshwater predator, the European catfish (*Silurus glanis*). Here we report how adults of Atlantic salmon are predated in the fishway of a large river of SW France, where the delayed and narrow passage created by the structure increases the probability of predator-prey encounter. We assessed predation risk by monitoring salmon and catfish in one fishway of the River Garonne, using video fish-counting from 1993 to 2016. We analysed the predation strategy of catfish using observations made with acoustic camera and RFID telemetry in 2016. Our results demonstrate a high predation rate (35%—14/39 ind.) on salmon inside the fishway during the 2016 spawning period migration. Our results suggest that a few specialized catfish individuals adapted their hunting behaviour to such prey, including their presence synchronized with that of salmon (i.e, more occurrences by the end of the day). Such results suggest that the spread of European catfish will potentially impact migration of anadromous species through anthropized systems.

## Introduction

The main causes of global Salmonid decline are well identified. Habitat fragmentation, habitat alteration, acidification and overexploitation seriously threaten populations of species such as the Atlantic salmon [1,2]. Furthermore, climate change, introduced fish species or predation are now considered as potential threats, but there is limited information on how these factors and their interactions will affect salmonid populations [2]. Introductions of large-bodied predator fish that forage at the apex of food webs are known to impact native fish populations and modify prey assemblages as well as food web structure [3,4]. A well-known example is given by the introduction of the Nile perch in African lakes that negatively impacted cichlid populations and the food web through top-down effects [5].

Largely introduced in the 1970s’ the European catfish *Silurus glanis* is now widespread in western and southern European freshwaters where it has established self-sustaining populations in most large rivers [6]. Large individuals can measure over 2.7 m total length and weigh 130 kg [7]. With its large gape size, the European catfish is a potential predator to many if not all native fishes, including anadromous species so that native species would no longer benefit from the size-refuge that protected them against native top-predators (e.g., pike; [8]).

In this context, human activities that affect fish movement may increase the exposure of Atlantic salmon to predators. Artificial structures (e.g., dams and weirs), even where equipped with fish passage devices, are suspected to reduced survival of prey fish species by increasing prey residence time and predator density [9] and therefore, encounter rates. Increased food resource availability, smaller passage width and and simplified structure inside fish ladders may trigger the emergence of trophic specialization among consumers [9,10]. Moreover, intraspecific variation in trophic specialization might explain the ability of introduced species to establish populations. In European catfish populations, some individuals have been observed to adapt their behaviour to forage on novel prey, leading to behavioural and trophic specialization [11].

The Atlantic salmon, considered as an endangered species in Western Europe, was eliminated in the mid 1900s from many large, heavily anthropized and fragmented rivers [12,13], such as the River Garonne (Southwest France). In the 1980s, a sustainable reintroduction plan and restoration programs to facilitate passage over obstacles were begun in the Garonne basin. The lower-most obstacle on the River Garonne (Golfech power plant) was therefore equipped in 1987 with a fish lift as fishway. The Atlantic salmon population was monitored since 1993 in the fishway where adult returns are counted with video [14]. However, potential predation by the European catfish inside the fishway is now raising concerns that introduced predators may challenge conservation efforts. The aim of this study is to assess the risk for the Atlantic salmon to be predated by the European catfish inside an anthropized system. To this end, we hypothesized that some specialized catfish individuals could adapt their foraging behaviour to this restricted and anthropized spatial environment leading to Atlantic salmon predation in the fishway.

## Materials and methods

### Study area

**Fig 1:**
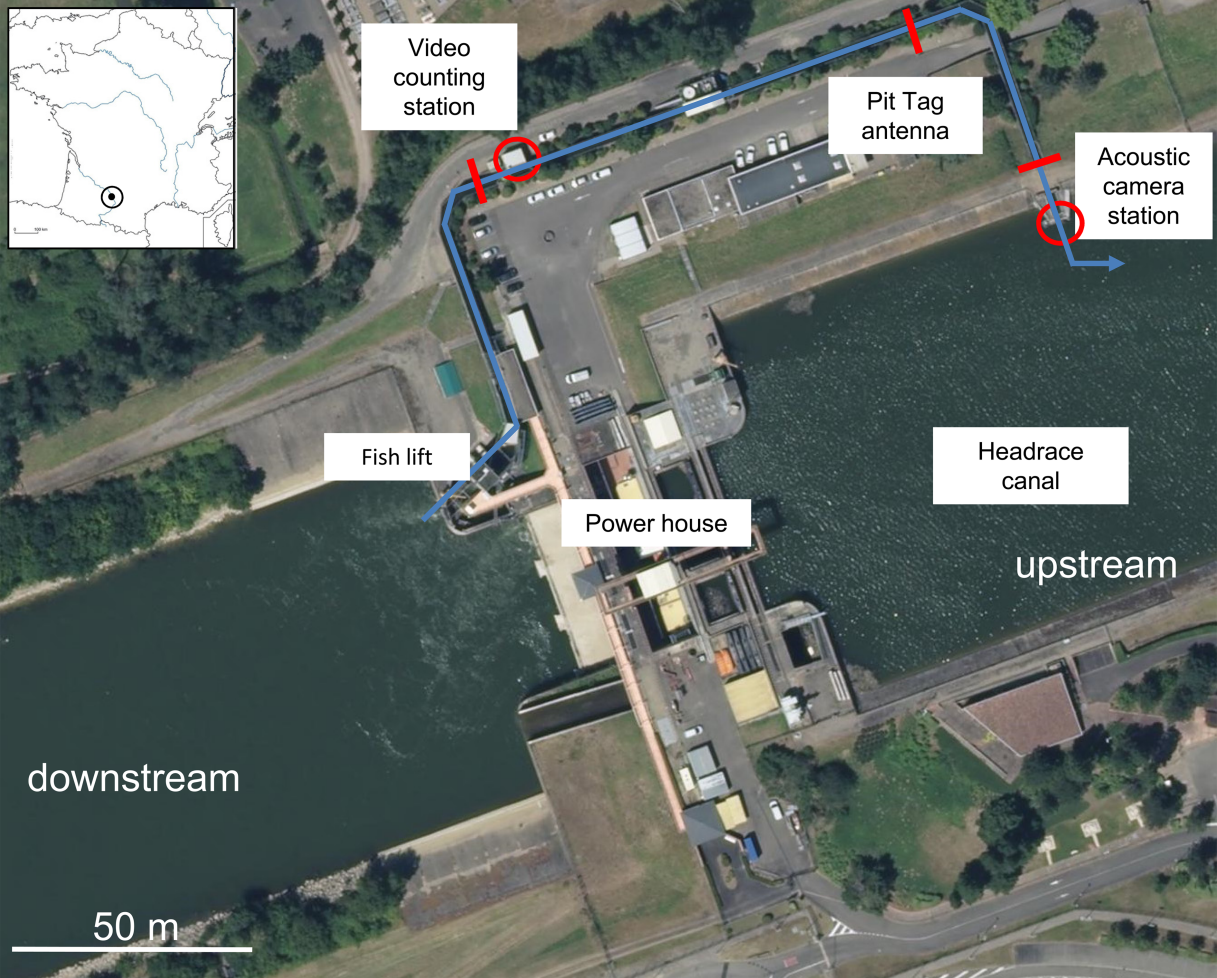
Location and overview of the Golfech fishway on the River Garonne.

Located in southwestern France, the Garonne River runs over 580 km from its source in the Pyrenees to the Atlantic Ocean. The Golfech–Malause hydroelectric complex was built in 1971 on the Garonne River (southwestern France) about 270 km from the river mouth (0°55’22.3”E; 44°06’37.6”N), downstream from the confluence with the Tarn River (Fig 1; see [15] for more details). This diversion-type hydropower facility is the first barrier for upstream migration of anadromous species in the Garonne River. The Golfech power plant structure was equipped in 1987 with a fish lift on the right bank of the tailrace. Fish are attracted into a 9-m long, 2.5-m wide and 1.5–4.5-m deep holding pool. At regular intervals (depending on fish passage frequency), fish are trapped and concentrated into a 3.3-m^3^ tank. This tank is raised (fish lift) and emptied upstream of the plant into a 250-m long, 2-m wide and 2.5-m deep transfer canal (Fig 1). Fish pass into this transfer canal before joining the headrace canal.

### Fish counting

A permanent video fish-counting station was installed in the fishway to monitor the number and timing of fish passage since 1993 (Fig 1). Migado, the association in charge of analysing these records, provided the daily numbers of net passages of European catfish and Atlantic salmon in front of the fish-counting station (i.e., for each fish species and each day, the number of upstream movements minus the number of downstream movements) between January 1^st^ 1993 and December 31^st^ 2016. The annual (or monthly) number of fish passing through the fishway was obtained by summing the daily net passages of the year (or month). We also obtained the number of catfish and salmon upstream and downstream passages for each hour from 2004 to 2016. We used these long-term data to describe the annual and seasonal timing of salmon and catfish and their behaviour in the fishway.

A temporary acoustic camera BlueView (Teledyne Blueview M900-2250 Dual Frequency series) was installed from the 4^th^ of April to the 26^th^ of May 2016 in the headrace canal at the upstream exit of the transfer canal in order to examine catfish and salmon behaviour in this unknown place where waters are deep and cloudy (Fig 1). The acoustic camera was placed at the exit of the fishway in order to the camera view range covered the area of the canal outlet (2.25 m wide per 1 m high) and therefore ensure that no fish could be missed. Moreover, specific morphological characteristics of Atlantic salmon and European catfish (body size and form, catfish head shape and salmon dorsal fin) can allow easily and undoubtedly distinguishing these two species from others. Salmon were counted at this place to be compared with salmon counted at the video counting station during the same period in order to estimate catfish predation on salmon inside the fishway. During this period, the transfer canal was emptied twice a week to ensure that no salmon remained in the fishway.

### European catfish tagging

A total of 35 European catfish were captured in the transfer canal in April 2015 (n = 10) and in April 2016 (n = 25) to be tagged in order to monitor their presence inside the fishway. Catfish were anaesthetised using a benzocaine solution at 10% (0.7 ml/l), measured and tagged with a 32 mm PIT tag (RI-TRP-WR2B, half duplex, 134 kHz, diameter 3.85 mm and weight 0.8 g in air; Texas Instruments). The procedure took less than 5 minutes. Fish were transferred to a tank of clean water to recover from anaesthesia and released just outside the upstream exit of the transfer canal. Fish tagging was ensured by Migado, the association in charge of monitoring fish at the fishway, in accordance with national legislation under the authorisation ‘Arrêté Préfectoral 2015–230’. Three antennae were fixed inside the transfer canal to detect tagged fish and to analyse the periods of catfish presence inside the fishway.

## Results

### Salmon and catfish numbers and timing

**Fig 2:**
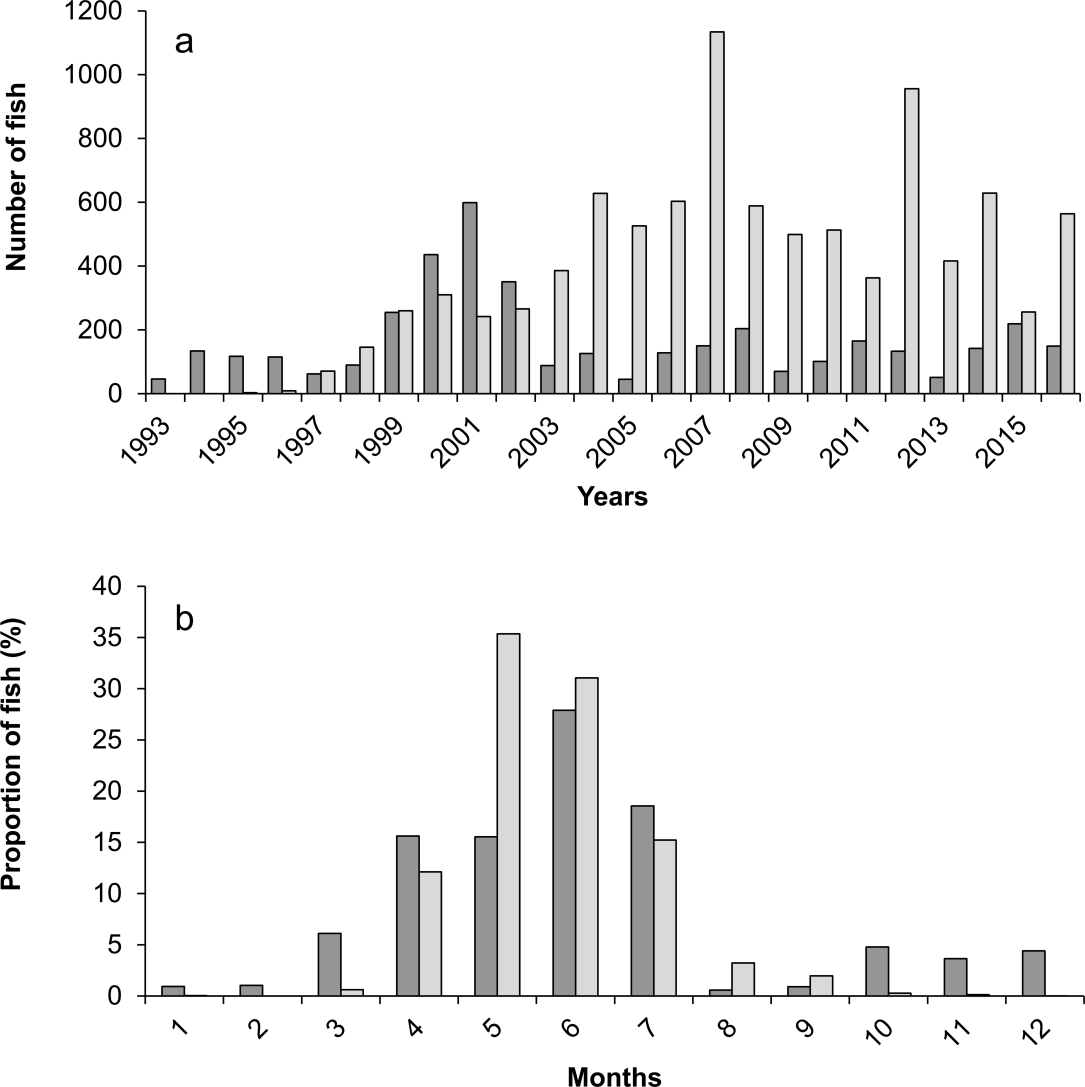
**Timings of passages of Atlantic salmon (dark grey) and European catfish (light grey) at the fishway in the River Garonne**: (a) annual net number of fish counted at the video fish-counting station since its installation in 1993; (b) month distribution (in %) of over the period 1993–2016.

The annual number of returning adult Atlantic salmon averaged 166 (±131 SD), ranging from a minimum of 45 individuals in 2005 to a maximum of 599 individuals in 2001 (Fig 2A). This number of Atlantic salmon exhibited a slight peak between 1999 and 2002. First European catfish passages at the video fish-counting station occurred in 1995, with three individuals. This number progressively increased until 2004 to reach an average of 590 (±232 SD) individuals per year during subsequent years. Years 2007 and 2012 exhibited the highest annual numbers of European catfish with 1134 and 956 individuals respectively (Fig 2A).

Between 1995 and 2016, nearly 95% of the European catfish passed between April and July with proportions reaching 35 and 31% in May and June (Fig 2B). The migration period of salmon is only slightly earlier with 78% of salmon passing between March and July. A small peak of salmon passages was first observed in autumn, but since 2003, this peak has disappeared and 96% of the salmon were observed to pass between March and July (data not shown).

**Fig 3:**
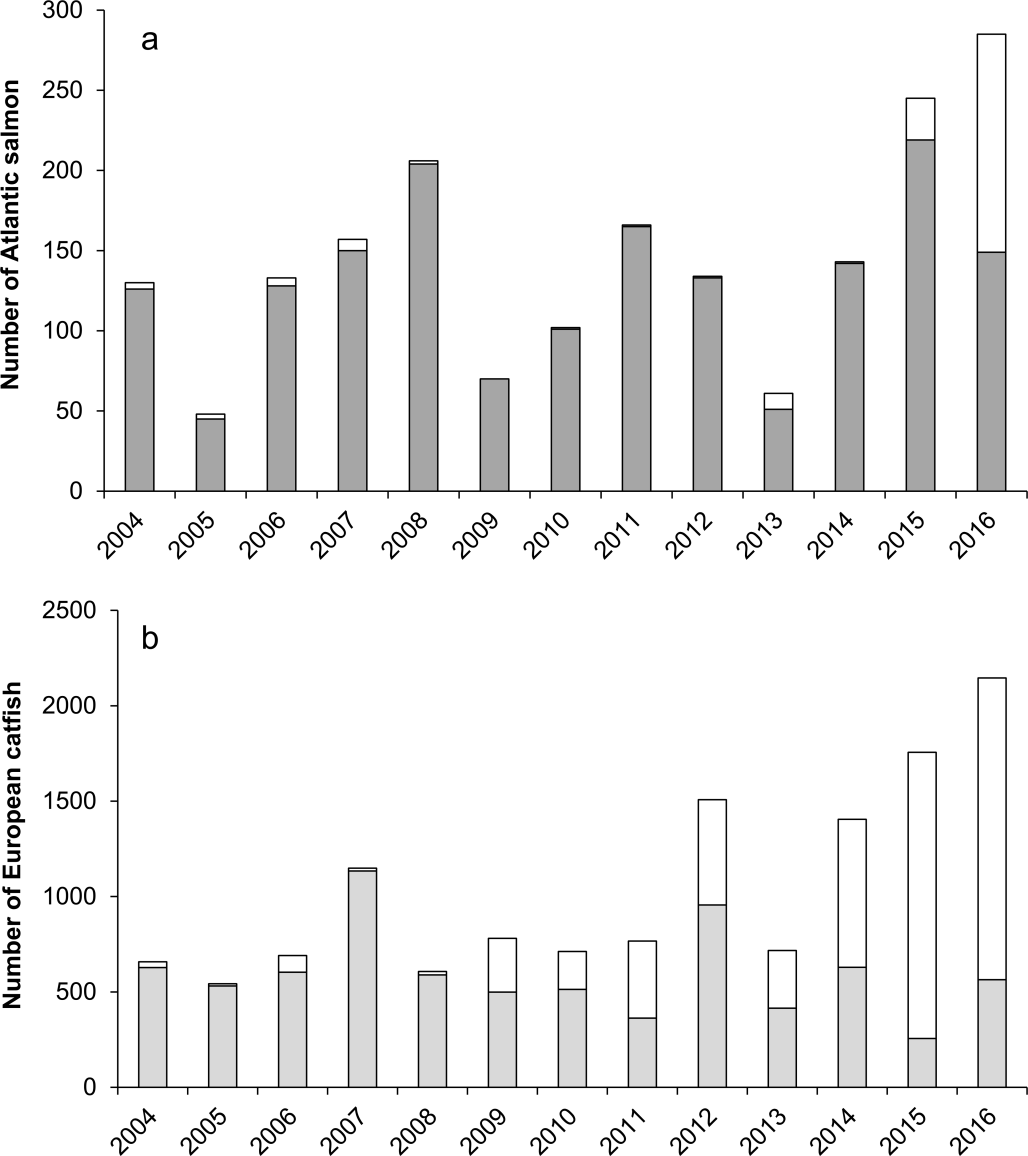
Number of Atlantic salmon (a) and European catfish (b) coming back in front of the video fish-counting station (in white) as compared with the annual net number of passages of Atlantic salmon (dark grey) and European catfish (light grey).

From 2004 to 2008, the frequency downstream passages by catfish was low. After 2009 it strongly increased, reaching in 2015 a frequency nearly six times higher than in previous year (Fig 3B). This suggests that catfish spent more and more time inside the fishway, going back and forth in front of the video fish-counting station, leaving and entering back the fishway and not only directly pass towards upstream. Similarly, the number of Atlantic salmon coming back in front of the video station was particularly high in 2016 as compared with previous years (Fig 3A).

**Fig 4:**
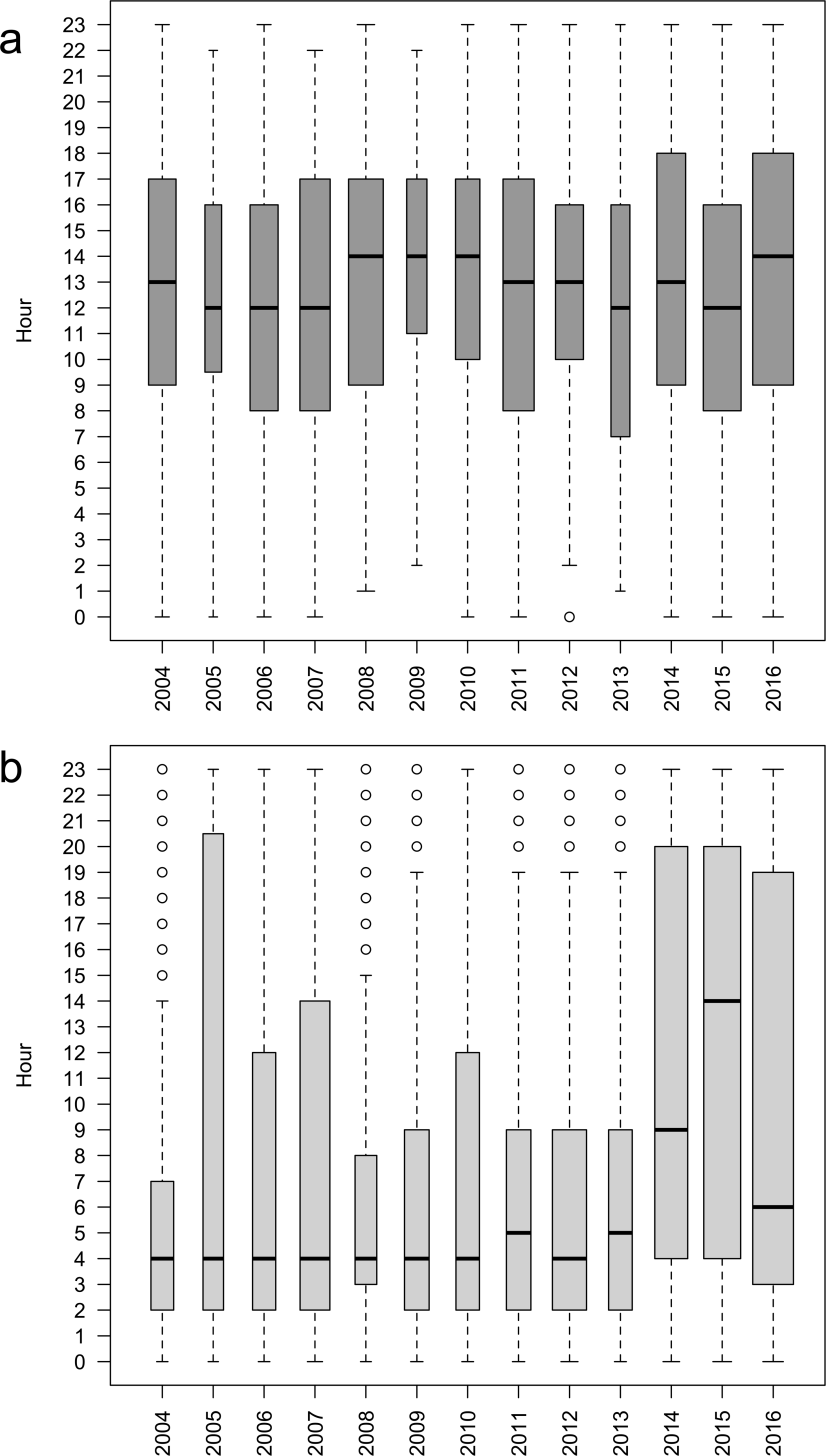
**Hourly timings of Atlantic salmon** (a) and European catfish (b) comings in front the video fish-counting station between 2004 and 2016.

Salmon preferentially came in front of the video fish-counting station during daytime between 8am and 9pm (median value around 1pm; Fig 4A). By contrast, catfish preferentially passed nightly between 11pm and 9am (median value at 4am). In 2014, 2015 and 2016, a higher proportion of European catfish were observed at the end of the day, between 5pm and 10pm (Fig 4B).

### European catfish visiting/occupancy in the fishway

**Fig 5:**
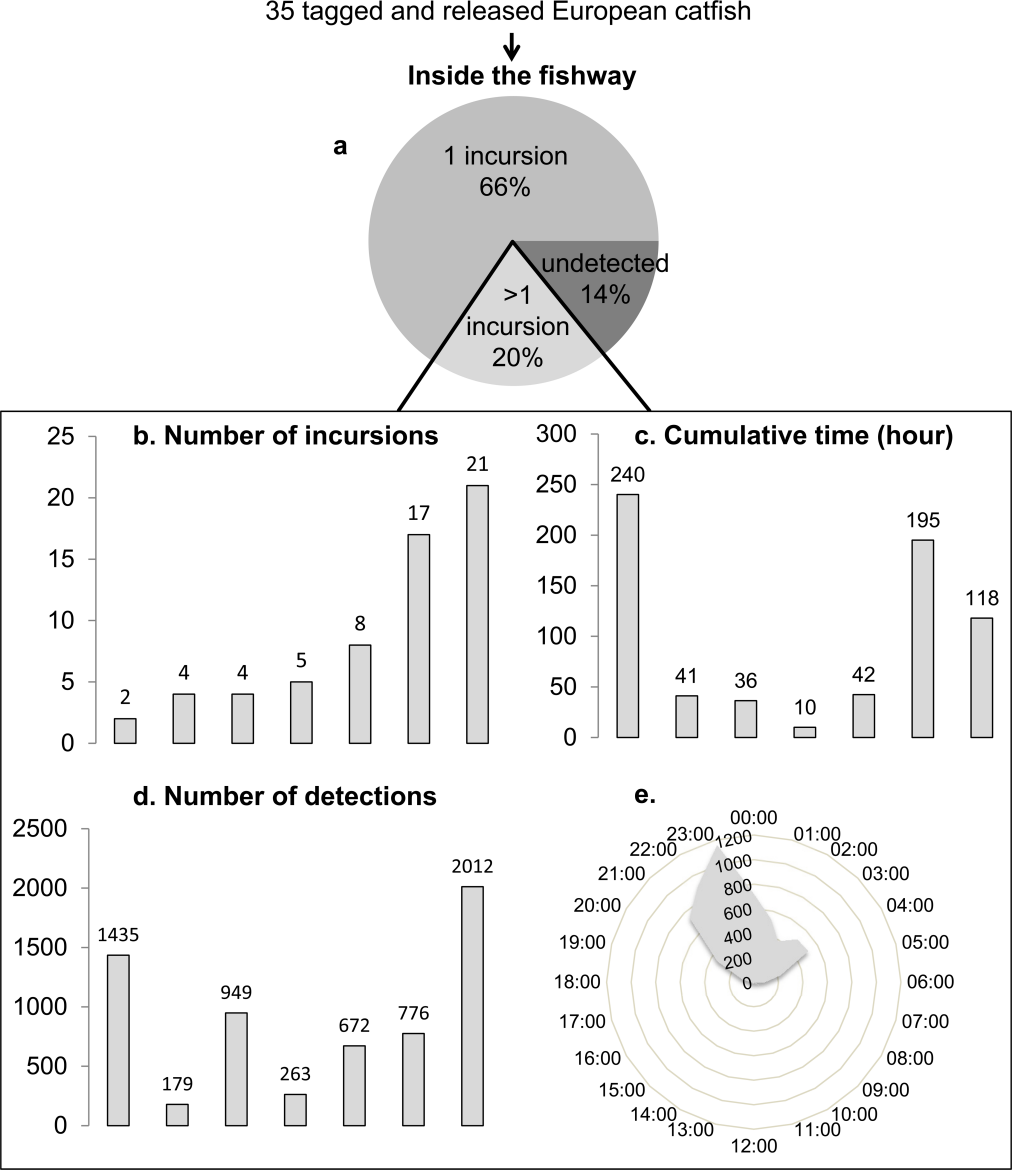
**European catfish visiting in the fishway**: (a) proportion of tagged individuals detected in the fishway and timings of the presence of the most active individuals (n = 7) in terms of (b) number of incursions, (c) number of detections by one of the antennae, (d) cumulative time spent and (e) hourly period of their presence in the fishway.

Detections occurred from April 20^th^ to July 14^th^, 2015 and from April 29^th^ to August 16^th^, 2016. Among the 35 catfish tagged and monitored, 30 (86%) were detected at least once by one of the antennae inside the transfer canal (Fig 5A). 23 catfish (66%) have performed only one annual incursion inside the transfer canal staying inside from less than 1 minute to 17.6 hours (Fig 5A). The other seven individuals (20%) have performed more than one incursion. The number of incursions they performed, the time of their incursions and the cumulative time they spent inside during the year of their release differed between individuals, with annual incursion number ranging from one to 21 (Fig 5B), annual number of detections ranging from 179 to 2012 (Fig 5C) and cumulative annual time inside ranging from 10 hours to 10 days (Fig 5D). These seven most active catfish individuals inside the fishway were detected at night, mainly at the beginning of the night from 9pm to 11pm (Fig 5E).

### European catfish predation on salmon

Salmon migration was observed at the video fish-counting station. Without catfish inside the fishway, direct upstream migration behaviour, with salmon swimming at the bottom of the fishway, was observed. With catfish inside, the migration behaviour was disrupted with many salmon going back and forth, swimming at the surface, staying for a long time inside before exiting, and sometimes being predated. See “S1 Movie” for direct predation of an adult salmon (80 cm total length) by a large European catfish (160 cm total length).

Acoustic camera records performed from April 4^th^ to May 26^th^ 2016 (51 days) at the exit of the fishway showed that there were often between one and six European catfish individuals waiting at the exit of the transfer canal. During this period, a total of 187 European catfish were observed exiting from the transfer canal and 86 coming back into the fishway.

During this same observation period (51 days), over the 39 salmon counted at the video fish-counting station, only 25 were observed at the exit of fishway by the acoustic camera. The remaining 14 salmon—unobserved with the acoustic camera (35%)—were predated by European catfish inside the transfer canal between the video station and the exit. None of them was detected in front of the video fish-counting nor at the exit of the fishway from 9pm to 2am. Among the 25 salmon that managed to exit, 12 (48%) spent less than 30 minutes, seven (28%) spent between 30 minutes and 1 hour, five (20%) spent between 1 h and 6 h and one (4%) spent near 14 h inside the transfer canal before exiting it. 18 of them (72%) were attacked by European catfish when exiting but none of the predation acts was successful. Over the 67 other individual fish of undetermined species that were observed exiting the transfer canal, 31 (46%) were attacked. Image resolution did not allow us to determine whether attacks on other smallest fish preys were successful or not.

## Discussion

Most Atlantic salmon populations are declining, conversely European catfish populations are increasing in western and southern European freshwaters. Despite ambitious rehabilitation plans, the Atlantic salmon population of the Garonne River remains very low especially since 2003. The European catfish was observed in the fishway of Golfech since 1997 with increasing passage numbers followed by an apparent stabilization since 2008.

The period of upstream migration of Atlantic salmon that mainly occurs from April to July coincides with the period of European catfish passages at the dam. The European catfish is not a migratory species but, as many other freshwater fish, upstream movements can be observed before the spawning period [16]. The seasonality of this behaviour, from April to July, is linked to warmer water temperature [17,18] and/or when more prey is available during the spring migration [19]. Indeed, the present observations proved that returning adults of Atlantic salmon are a prey for the European catfish in the River Garonne. In a preliminary study, using DNA metabarcoding, Guillerault et al [20] have found DNA of Atlantic salmon in catfish faeces in the River Garonne, but without certainty that prey were healthy and not already weak or dead. Here, we observed predation acts and we report that 35% of the 40 migrating salmon observed at the dam here were consumed inside the fishway.

Predation inside the fishway is high, despite the observed mismatch between daytime activities of both species (diurnal for Atlantic salmon, nocturnal for European catfish) that should limit the predation risk. Individual tagging suggested that predation was due to catfish individuals that were staying at the exit, experiencing upstream and downstream movements inside the fishway and/or anticipating comings inside the fishway at the end of the day to increase the probability to encounter salmon inside. Slavík et al [18] demonstrated that the European catfish in a river is not strictly nocturnal in activity. The same authors demonstrated, from electromyogram biotelemetry records, that there is considerable individual variability in diel activity depending on different individual behaviour and ability to use energy reserves [21]. Furthermore, the European catfish is known to display individual trophic specialisation through foraging on terrestrial birds by intentional beaching [11]. Diet plasticity is a common phenomenon in top predators with high-energy requirements and the ability to learn to utilize new resources. As a long lifespan species with novel behaviours—massive aggregations [22], beaching [11], predation in fishway (this study)—European catfish is distinct from many other freshwater fish, and exhibits adaptations to its environment that are likely to contribute to its invasive success.

Here, the anthropization of the river provides a local opportunity for European catfish to exploit migrating Atlantic salmon. The continuous occurrence of several catfish individuals at the exit of the fishway suggests that predators occupy this strategic location to capture other fish prey. Indeed, unsuccessful predation acts on Atlantic salmon observed by acoustic camera at the exit of the fishway suggest that Atlantic salmon escape more easily than other fish from catfish predation thanks to its high-speed swimming performance. Further investigation would be necessary to demonstrate selectivity or opportunism to consume prey. Our preliminary results show that 46% (31/67) of undetermined freshwater fish were attacked compare to 72% (18/25) for salmon. Large adult salmon (mean 80 cm total length), compare to generally smaller freshwater fish prey, could be preferentially selected by large catfish.

Multiple stressors are known to interact synergistically to amplify the individual effects of global change drivers on species and ecosystems [23,24,25]. The results suggest that the presence of dams (and fishways) and a new predator have additional impacts on the precarious Atlantic salmon population of the River Garonne. In complex narrow fishways, introduced European catfish can ambush and predate their prey, thus amplifying the ecological consequences of an anthropogenic perturbation [9].

Due to human introduction coupled with future climate change, the range extension of the European catfish, especially in the north of Western Europe, will continue. In this context, even if no strong impact may appear for freshwater fish [6], the potential future risks for large anadromous species should be taken into account. Indeed large anadromous fish species may be more sensitive to catfish than other freshwater fish that do not need to use the fishway. Conversely to other freshwater species, large anadromous species may have not developed defence strategies against predator before catfish establishment. Moreover, by predating reproductive adults, the impact of catfish is certainly stronger on anadromous life cycle. The potential novel predation pressure on these non co-evolved prey, coupled with increased human activity (e.g. dam, fisheries), is concerning, especially in the case of declining populations of anadromous species.

## Supporting information

S1 Movie Adult Atlantic salmon have a new freshwater predator. (MP4)
